# Occupational Specific Heavy Metal Exposure: Association With Age and Anthropometric Characteristics Relative to Hypertension

**DOI:** 10.1155/ijhy/5085833

**Published:** 2026-10-18

**Authors:** Godwin Chinedu Uzomba, Collins Nwafor, Chibuike Ugah, Henry Ogbodo, Chukwuka Amaku, Ken Onwe, Tamuno-Ibelema Cookey-Gam, Godwin Sunday Obaje, Ejikeme Mbajiorgu

**Affiliations:** ^1^ Department of Anatomy Faculty of Basic Medical Sciences College of Medical Sciences Alex Ekwueme Federal University Ndufu-Alike Abakaliki Ebonyi Nigeria cmsnepal.edu.np; ^2^ Department of Anatomical Sciences School of Biomedical Sciences Faculty of Health Sciences University of Witwatersrand Johannesburg South Africa wits.ac.za

**Keywords:** anthropometric characteristics, blood pressure, heavy metals, occupational groups, regression analysis

## Abstract

The present study aimed at investigating the association between serum heavy metals, anthropometric characteristics, and hypertension among different occupations and analyzing the prediction between heavy metals, anthropometrics, and hypertension among male and female miners, artisans, laborers, and farmers. Body weight, height, and circumferences were measured via the direct standard anthropometric approach. Blood pressure (BP) was measured using a standardized mercury sphygmomanometer with an appropriate cuff size. Blood samples were analyzed using an atomic absorption spectrophotometer. Chi‐square and binary logistic regression analysis were conducted at a significance level of *p* < 0.05 (^∗^). For miners, there was no significant association between serum heavy metals, anthropometric characteristics, and hypertension (*p* > 0.05). Among artisans, age and anthropometric circumferences showed significant association with hypertension (*χ* ^2^ = 5.266 – 21.206, *p* < 0.05^∗^). Among laborers, age was significantly associated with hypertension (*χ* ^2^ = 62.548, *p* < 0.05∗). For farmers, age showed significant association with hypertension (*χ* ^2^ = 3.925, *p* < 0.05∗). The derived regression model, which included age as a predictor, showed that increasing age was significantly associated with higher odds of hypertension among miners (aOR: 1.076, 95% CI: 1.029–1.125) and artisans (aOR: 11.938, 95% CI: 3.349–38.621). Furthermore, anthropometric circumferences were significant predictors of hypertension among artisans (aOR: 3,427, 95% CI: 1.205–9.747) and laborers (aOR: 2.50, 95% CI: 1.130–3.207). The study contributes to the understanding of relevant of heavy metals and specific anthropometrics to BP. Understanding the effects of heavy metals and age have implications in occupational health and contribute to the development of public health interventions.

## 1. Introduction

Hypertension is an established chronic noncommunicable disease (NCD), affecting about 1.28 billion adults globally. Hypertension is associated with severe health complications affecting the heart, brain, and kidneys, resulting in heart attack, heart failure, stroke, kidney damage, and vision problems. It is one of the important cardiovascular disease risk factors that affect roughly 30% of adult population without gender specificity [1, 2] and a major source of premature death and disability globally [3].

Environmental heavy metal pollution is an important public health concern, and elevated serum levels have been associated with increased risk of hypertension [4] and other cardiovascular diseases, though this has not received much attention. Though reports indicate that heavy metal exposure could contribute more to hyeprtension than lifestyle risk factors, including physical inactivity, alcohol consumption, obesity, and unhealthy diets [5–9]. However, some essential trace elements, like iron, zinc, and selenium, are beneficial to various biophysiological processes and within certain safe ranges and play essential roles in various body enzymatic reactions that are critical in blood pressure (BP) regulations [10].

The alarming increase in hypertensive individual in the society is highly mind burgling and raises the curiosity on the role of occupational heavy metal exposure levels, this, on the backdrop of the fact that modern society and economy are occupationally structured [11, 12], with about 93 per cent of the global informal employment in developing countries [13]. The number of people with hypertension has doubled since 1990, and 1.28 billion adults worldwide are currently hypertensive [1]. While epidemiological data suggested a potential relationship between environmental metal exposure and hypertension [14], the link to occupational exposure is yet to be fully explored.

Moreover, associated with occupational exposure is human and industrial development and advancement, which has led to industrial products such as paints, cosmetics, pesticides, and herbicides, commonly used in agricultural, government, and industrial work spaces, as well as in homes. Exposure to these chemicals and to contain trace amounts of heavy metals in the workplace greatly contribute to occupational and environmental exposure and overall health risks [15]. Reports show that both short‐ and long‐term exposures to heavy metals (lead and cadmium) lead to their bioaccumulation in the body and above certain limits, resulting in interferences with BP regulations with consequent elevation, alterations of biochemical processes, altered cellular and humoral immune responses, and an increased risk of hypertension [16–20].

Amongst other toxicities such as endothelial dysfunction, disruption of homeostasis, and generation of reactive oxygen species (ROS) leading to derangements in cellular processes such as cell growth, proliferation, survival, and metabolism, thereby causing oxidative damage [21], heavy metals interact with DNA and nuclear proteins, inducing damage and conformational changes that may lead to hypertension [7, 21–23].

A report by Nawrot et al. [24] indicated that a twofold increase in blood lead levels was associated with a rise of 1–1.25 mmHg in systolic blood pressure (SBP) and 0.6 mmHg in diastolic blood pressure (DBP), while Kisok and Park [25] reported that higher blood concentrations of lead (Pb) and cadmium (Cd) were positively associated with elevated BP and an increased prevalence of hypertension among adults in South Korea. Additionally, Wang et al. [26] observed that elevated blood Pb levels were correlated with BP in hypertensive patients.

Correspondingly, a progressive increase in BP has been associated with an increased adipose tissue [27]. Moreover, studies have documented correlation between body mass index (BMI), Waist‐to‐Hip ratio (WHR), Waist‐to‐Height ratio (WHtR), and BP [28–32]. These anthropometric indices are crucial risk factors for various chronic diseases, including hypertension [33], and Type 2 diabetes mellitus [34]. Thus, fat accumulation in the chest can increase chest wall thickness, leading to heightened cardiovascular resistance, compromised blood circulation, and altered BP [35]. Consequently, Nghia et al. [36] observed a relationship between fat accumulation in abdominal organs and elevated BP. Furthermore, Ezemagu et al. [37] noted that variations in SBP and DBP were dependent on BMI, WHtR, and waist and hip circumference, but not on WHR in both sexes. Additionally, a positive association between WHtR and the incidence of hypertension was observed among Korean adults [38]. Their findings suggest that estimating the trio‐anthropometric predictors could serve as a useful tool for routine screening or preliminary diagnosis of hypertension. The primary anthropometric contributor to hypertension is an imbalance between caloric intake and energy expenditure [39]. For instance, caloric intake may sometimes contain significant quantities of heavy metals, which accumulate in the body and contribute to adverse health outcomes such as cardiovascular diseases and hypertension [40].

Individuals in various occupations are susceptible to heavy metal exposure through inhalation and contaminated drinking water, food, or dermal contact, posing serious health risk [23, 41]. Industrial products, processes, and oil drilling have led to elevated levels of heavy metals in drinking water worldwide [22], especially in the riverine areas of Nigeria where there has been ongoing oil drilling for decades. Furthermore, occupations such as miners, artisans, laborers, and farmers are at risk of exposure to lead and cadmium via various pathways, including contaminated dust particles, sculptural imprinting, and grinding process [42]. Once in contact with biological tissues, heavy metals act as systemic toxins, interacting with specific biophysiological systems leading to alteration of functional and ultimately causing hypertension, cardiotoxicity, or teratogenic effects [43, 44]. Moreover, the United Nation Environment Program [45] observed that mining activities involving heavy metals without adequate containment strategies facilitate contamination of the food chain through bioaccumulation in plants and water, posing harmful effects upon consumption.

Human exposure to metals is inevitable, and studies have reported gender differences in metal toxicity resulting in the disruption of cellular organelles and processes [25], reproductive incapacity, [46, 47], and cancer induction [22], amongst others. While the association between hypertension and occupational exposure to lead and cadmium remains inconclusive, some studies have reported correlation between hypertension and heavy metals exposure in occupational settings [48, 49], thereby highlighting the need for more studies on this subject on the backdrop of certain occupations (commonly considered safe) but nonetheless are sources of heavy metal exposure. Such studies will help to better evaluate the relationship between heavy metal exposure and hypertension across these different occupations.

This study, therefore, investigated the relationship between some occupationally related serum heavy metals and BP, relative to some trio anthropometric index (BMI), WHR, and WHtR characteristics and determined a predictive model for hypertension based on serum heavy metals and these anthropometric characteristics, while accounting for sex differences.

## 2. Methods

### 2.1. Study Area

The study was conducted in the Ikwo‐Abakaliki region of Ebonyi State, which has an estimated population of 1,200,000 inhabitants and is notably contaminated with heavy metals such as lead and cadmium due to heavy mining activities. Located within the moist semideciduous forest zone, the region contains several open pit mines contributing to air, water, and soil pollution. It must be noted that this study was conducted during the peak period of mining activities.

### 2.2. Participants

The study involves 538 participants, comprising the following.

Miners (57 males: age: 35.65 ± 10.64 years, weight: 69.79 ± 12.04 kg, and height: 1.70 ± 0.07 m, with serum levels of lead: 16.13 ± 3.05 μg/dL and cadmium: 3.25 ± 0.57 μg/L; and 53 females: age: 30.47 ± 11.26 years, weight: 62.11 ± 13.03 kg, and height: 1.60 ± 0.09 m, with serum level of lead: 25.42 ± 4.11 μg/dL and cadmium: 3.11 ± 0.84 μg/L).

Artisans (69 males: age: 33.35 ± 8.78 years, weight: 68.80 ± 9.56 kg, and height 1.70 ± 0.06 m, containing serum level of lead: 16.02 ± 6.50 μg/dL and cadmium: 2.69 ± 0.50 μg/L; and 57 female: age: 27.35 ± 9.96 years, weight: 58.86 ± 11.12 kg, and height: 1.60 ± 0.07 m, with serum level of lead: 17.67 ± 4.22 μg/dL and cadmium: 1.95 ± 0.89 μg/L).

Laborers (83 males: age: 30.16 ± 10.67 years, weight: 60.41 ± 14.23 kg, height: 1.62 ± 0.09 m, lead: 16.32 ± 5.16 μg/dL, and cadmium: 3.89 ± 0.72 μg/L; and 69 females: age: 31.10 ± 9.21 years, weight: 67.13 ± 15.75 kg, height: 1.70 ± 0.09 m, lead: 23.45 ± 4.66 μg/dL, and cadmium: 2.85 ± 0.90 μg/L).

Farmers (75 males: age: 31.43 ± 11.0 years, weight: 63.17 ± 9.59 kg, height: 1.69 ± 0.10 m, lead: 10.97 ± 2.88 μg/dL, and cadmium: 2.62 ± 0.62 μg/L; and 75 females; age: 30.13 ± 9.95 years, weight: 60.83 ± 14.12 kg; height: 1.61 ± 0.08 m, lead: 16.06 ± 2.73 μg/dL, and cadmium: 2.66 ± 0.69 μg/L).

Participants were recruited from occupational groups working in areas with potential exposure to heavy metals. Eligible participants were identified at their respective workplaces and invited to participate in the study. Participants were included using convenience sampling techniques. The selection of these categories of occupations was based on their predominant nature within the selected study area/community.

### 2.3. Research Design

This study implemented a cross‐sectional design and was conducted in a mining setting. Two types of data were obtained in different ways: first, individual anthropometrics and BP were collected by the researchers; second, blood samples were collected by two trained medical laboratory scientists.

### 2.4. Data Collection

Baseline anthropometric indices such as the BMI, WHR, and WHtR were measured. Height and weight were measured with the aid of a stadiometer (model RGZ‐160, Jiangsu Suhong Medical Instruments Co., Ltd, Shanghai, China). A nonstretchable anthropometric tape (SECA‐200, SECA, Hamburg, Germany) was used to measure circumferences (waist circumference [WC] and hip circumference). WC was measured in the horizontal plane at the midpoint between the anterior iliac crest and the inferior margin of the rib. WC was further classified into normal (men: ≤ 102 cm, female: ≤ 88 cm) and high (men: ≥ 102 cm, female: ≥ 88 cm) [50, 51], and hip circumference was measured at the widest part of the buttocks, as previously published method [37, 52], and was classified as normal (men: 94–105 cm, women: 97–108 cm) or high (men: > 105 cm, women: ≥ 108 cm) [53]. Both circumferences were measured to the nearest 0.1 cm. From these data, BMI, WHR, and WHtR were calculated and classified as follows: WHR was classified as low (men: ≤ 0.90, women: ≤ 0.80) or high (men: ≥ 0.90, women: ≥ 0.80) [53, 54]. WHtR was classified as healthy (men/women: 0.40–0.49) or high (men/women: > 0.50) [55].

SBP and DBP were measured on the right arm using a standardized mercury sphygmomanometer (Baumanometer, Copiague, NY) with an appropriate cuff size. BP was measured three times at 5‐min rest intervals between measurements, and the mean values were calculated and used for analysis. Hypertension is confirmed if the SBP was ≥ 140 mmHg and/or DBP was ≥ 90 mmHg [56]. Pulse pressure (PP) was calculated as the difference between SBP and DBP.

A total of 5 mL of blood was collected from each participant by two certified hematologists into a plain, siliconized glass tube. The blood samples were allowed to clot for 10 min and were subsequently centrifuged for 15 min to obtain the serum [38]. Additionally, the serum samples were carefully decanted and aspirated into sterile plain tubes and subsequently stored in ice pack until analysis. The venipuncture site was cleaned using cotton wool soaked in 70% alcohol before blood collection. All laboratory materials used for sample preparation were sterilized using autoclave (model type: ST 2228; rated at 230v 50 Hz) at a temperature of 125^o^C. Reagent blanks and quality‐control procedures were also included to monitor potential contamination during the analytical process. All samples were anonymized to prevent bias and analyzed using a Graphite Furnace Atomic Absorption Spectrophotometer (GFAAS). Serum samples were treated with a dilute matrix modifier or acid solution to precipitate proteins and release bound metal ions. Sample deproteinization was performed using 5% HNO_3_ to minimize inference from organic matrix. The calibration working range was adjusted to the concentrations expected in the occupational samples. Calibration curves for lead (Pb) and cadmium (Cd) were prepared using five concentration points for each element, along with a reagent blank. Before calibration, blood samples were analyzed to determine the endogenous concentrations of the target metals. Several series of calibration standards prepared in the blood matrix were stored in a freezer (< 20^0^) until analysis [57, 58]. The analytical wavelengths were set at 283.3 nm for Pb and 228.8 nm for Cd, with a lamp current of 10 mA and 4 mA for Pb and Cd, respectively. The blood samples were quantified as described by Batra et al. [59]. Lead (Pb) concentrations were classified as normal (5–10 μg/dL) and abnormal (> 10 μg/dL), while cadmium (Cd) concentrations were classified as normal (5 μg/L) and abnormal (> 5 μg/L) [59, 60]. The results of the metal analysis were corrected using reagent blanks, as described in the referenced protocol [16].

### 2.5. Statistical Analysis

The data were analyzed via SPSS Version 23 (IBM, USA). Normality was assessed via Kolmogorov–Smirnov test, and subsequent statistical tests were selected accordingly. Percentage distribution of hypertension across different occupations exposed to heavy metals was considered. Descriptive statistics were presented as mean ± standard deviation (SD). The univariate analysis of the associations between anthropometric characteristics, heavy metals, and hypertension was performed. A logistic regression model was used to assess the associations between anthropometric characteristics, heavy metals, and hypertension. Significant level of *p* < 0.05 was set a priori for all tests.

## 3. Results

The percentage distribution of hypertension across different occupations exposed to heavy metals is presented in Figure 1. The result shows that the serum blood level of lead was significantly greater in females that that in males across all the different occupations evaluated, but the level of cadmium showed slight fluctuations across all the occupational categories. Also, while the level of lead peaked in female miners, followed by the female laborers, the highest serum cadmium level was recorded in male laborers.

**FIGURE 1:**
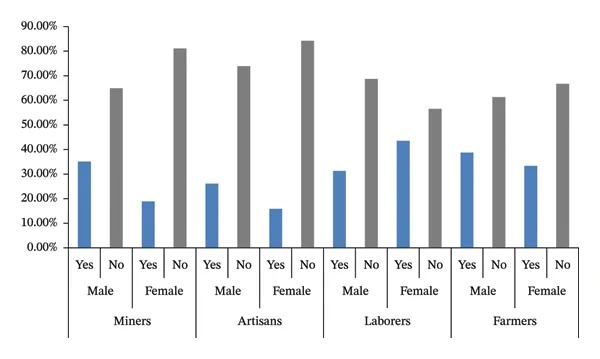
Percentage distribution of hypertension across different occupation exposed to heavy metals. “Yes,” signifies presence of hypertension, while “No,” signifies absence in both males and females.

Descriptive results are presented in Table 1. A total of 11 variables were tested in relation to hypertension across four occupational groups (i.e., miners, artisans, laborers, and farmers) in two sex groups (i.e., males and females). There was no significant association between hypertension and the exposed variables (Pb and Cd) among miners, artisans, laborers, and farmers (Table 2). However, significant associations were observed between hypertension and age and anthropometric circumferences among artisans. Furthermore, age was significantly associated with hypertension among laborers and farmers.

**TABLE 1:** Anthropometrics, blood pressure, serum heavy metals, and descriptive results as mean ± standard deviation (SD) at confidence intervals (95% CI) for males and females.

Occupations	Parameters	Male	Females
		Mean ± SD	Mean ± SD
Miners	Age (years)	35.65 ± 10.64	30.47 ± 11.26
	Weight (kg)	69.79 ± 12.04	62.11 ± 13.03
	Height (m)	1.70 ± 0.07	1.60 ± 0.09
	BMI (kg/m^2^)	24.20 ± 3.66	24.40 ± 5.02
	WC (cm)	84.70 ± 9.04	86.09 ± 11.21
	HC (cm)	97.82 ± 8.82	97.87 ± 9.90
	WHR	0.87 ± 0.04	0.88 ± 0.05
	WHtR	50.05 ± 5.37	55.40 ± 7.27
	SBP (mmHg)	128.77 ± 15.33	119.72 ± 14.72
	DBP (mmHg)	80.28 ± 11.57	72.87 ± 12.46
	Pulse pressure	48.49 ± 10.67	45.68 ± 8.86
	Lead (μg/dL)	16.70 ± 5.41	22.32 ± 6.43
	Cadmium (μg/L)	3.25 ± 0.57	3.11 ± 0.84

Artisans	Age (years)	33.35 ± 8.78	27.35 ± 9.96
	Weight (kg)	68.80 ± 9.56	58.86 ± 11.12
	Height (m)	1.70 ± 0.06	1.60 ± 0.07
	BMI (kg/m^2^)	23.81 ± 2.65	22.96 ± 4.07
	WC (cm)	82.43 ± 8.37	82.91 ± 10.08
	HC (cm)	95.13 ± 6.59	94.18 ± 7.25
	WHR	0.87 ± 0.05	0.88 ± 0.07
	WHtR	48.46 ± 4.44	51.89 ± 6.73
	SBP (mmHg)	130.32 ± 12.41	123.82 ± 15.85
	DBP (mmHg)	81.43 ± 8.02	76.09 ± 11.63
	Pulse pressure	50.67 ± 7.46	49.07 ± 11.99
	Lead (μg/dL)	16.02 ± 6.50	17.67 ± 4.22
	Cadmium (μg/L)	2.69 ± 0.50	1.95 ± 0.89

Laborers	Age (years)	30.16 ± 10.67	31.10 ± 9.21
	Weight (kg)	60.41 ± 14.23	67.13 ± 15.75
	Height (m)	1.62 ± 0.09	1.70 ± 0.09
	BMI (kg/m^2^)	22.55 ± 3.33	22.89 ± 3.09
	WC (cm)	81.75 ± 13.32	79.29 ± 10.56
	HC (cm)	96.92 ± 16.45	92.13 ± 10.21
	WHR	0.83 ± 0.07	0.86 ± 0.06
	WHtR	50.58 ± 8.79	46.70 ± 6.21
	SBP (mmHg)	126.69 ± 15.27	132.43 ± 12.79
	DBP (mmHg)	81.64 ± 10.63	85.07 ± 8.56
	Pulse pressure	43.80 ± 8.75	45.57 ± 9.53
	Lead (μg/dL)	16.32 ± 5.16	23.45 ± 4.66
	Cadmium (μg/L)	3.89 ± 0.72	2.85 ± 0.90

Farmers	Age (years)	31.43 ± 11.0	30.13 ± 9.95
	Weight (kg)	63.17 ± 9.59	60.83 ± 14.12
	Height (m)	1.69 ± 0.10	1.61 ± 0.08
	BMI (kg/m^2^)	22.24 ± 3.44	23.02 ± 3.19
	WC (cm)	80.55 ± 10.79	81.07 ± 12.94
	HC (cm)	92.13 ± 10.54	105.53 ± 9.43
	WHR	0.86 ± 0.06	0.84 ± 0.07
	WHtR	47.80 ± 6.64	50.47 ± 8.29
	SBP (mmHg)	132.97 ± 12.85	130.23 ± 14.02
	DBP (mmHg)	85.84 ± 8.64	83.43 ± 10.64
	Pulse pressure	46.04 ± 8.84	45.87 ± 8.49
	Lead (μg/dL)	10.97 ± 2.88	16.06 ± 2.73
	Cadmium (μg/L)	3.03 ± 1.15	3.01 ± 1.18

**TABLE 2:** Univariate analysis for the association between anthropometric characteristics, heavy metals, and hypertension.

Occupation	Variable		Hypertension (%)	Normotensive (%)	OR	*p* value
Miners	Sex	Male	20 (66.7)	37 (46.8)	0.440	0.064
		Female	10 (33.3)	42 (53.2)		
	Age	Young	1 (33.3)	9 (11.4)	0.886	0.540
		Adult	29 (66.7)	70 (88.6)		
	BMI	Nonobese	24 (80.0)	71 (89.9)	2.219	0.169
		Obese	6 (20.0)	8 (10.1)		
	WC	Normal	22 (73.3)	64 (81.0)	0.645	0.380
		High	8 (20.0)	15 (19.0)		
	HC	Normal	10 (33.3)	38 (48.1)	0.539	0.165
		High	20 (66.7)	41 (51.9)		
	WHR	Low	17 (56.7)	33 (41.8)	1.823	0.163
		High	13 (43.3)	46 (58.2)		
	WHtR	Healthy	12 (40.0)	31 (39.2)	0.969	0.942
		High	18 (60.0)	48 (60.8)		
	Lead	Normal	5 (16.7)	13 (16.5)	1.015	0.979
		Abnormal	25 (83.3)	66 (83.5)		
	Cadmium	Normal	3 (11.1)	73 (92.4)	0.431	0.120
		Abnormal	27 (88.9)	6 (7.6)		

Artisans	Sex	Male	18 (66.7)	51 (51.5)	0.531	0.161
		Female	9 (33.3)	48 (48.5)		
	Age	Young	10 (37.0)	81 (81.8)	0.131	**< 0.001**
		Adult	17 (63.0)	18 (18.2)		
	BMI	Nonobese	17 (63.0)	78 (78.8)	0.458	0.091
		Obese	10 (37.0)	21 (21.2)		
	WC	Normal	22 (81.5)	94 (94.9)	0.234	**0.022**
		High	5 (18.5)	5 (5.1)		
	HC	Normal	23 (85.2)	98 (99.0)	0.059	**0.001**
		High	4 (14.8)	1 (1.0)		
	WHR	Low	13 (48.1)	63 (63.6)	0.531	0.145
		High	14 (51.9)	36 (36.4)		
	WHtR	Low	14 (48.1)	37 (37.4)	0.643	0.310
		High	13 (51.9)	62 (62.6)		
	Lead	Normal	1 (5.70)	4 (4.0)	0.960	0.288
		Abnormal	26 (96.3)	95 (96.0)		
	Cadmium	Normal	24 (88.9)	82 (82.8)	1.659	0.445
		Abnormal	3 (11.1)	17 (17.3)		

Laborers	Sex	Male	26 (46.4)	57 (59.4)	1.686	0.122
		Female	30 (53.6)	39 (40.6)		
	Age	Young	1 (1.8)	10 (10.4)	0.156	**0.007**
		Adult	55 (98.2)	86 (89.6)		
	BMI	Nonobese	1 (1.8)	3 (3.1)	0.564	0.619
		Obese	55 (98.2)	93 (96.9)		
	WC	Normal	32 (57.1)	73 (76.0)	0.420	0.015
		High	24 (42.9)	23 (24.0)		
	HC	Normal	29 (51.8)	45 (46.9)	1.217	0.559
		High	27 (48.2)	51 (53.1)		
	WHR	Low	34 (60.7)	72 (75.0)	0.515	0.064
		High	22 (39.3)	24 (25.0)		
	WHtR	Healthy	23 (41.1)	37 (38.5)	1.111	0.758
		High	33 (58.9)	59 (61.5)		
	Lead	Normal	12 (21.4)	24 (25.0)	0.818	0.617
		Abnormal	44 (78.6)	72 (75.0)		
	Cadmium	Normal	47 (83.9)	82 (85.4)	0.892	0.805
		Abnormal	9 (16.1)	14 (14.6)		

Farmers	Sex	Male	29 (54.7)	46 (47.4)	0.746	0.393
		Female	24 (45.3)	51 (52.6)		
	Age	Young	3 (5.7)	17 (17.5)	0.282	**0.041**
		Adult	50 (94.5)	80 (82.5)		
	BMI	Nonobese	46 (86.8)	83 (85.6)	0.902	0.836
		Obese	7 (13.2)	14 (14.4)		
	WC	Normal	44 (83.0)	84 (86.6)	0.757	0.554
		High	9 (17.0)	13 (13.4)		
	HC	Normal	28 (52.8)	59 (60.8)	0.721	0.343
		High	25 (47.2)	38 (39.2)		
	WHR	Low	24 (45.3)	53 (54.6)	0.687	0.273
		High	29 (54.7)	44 (45.4)		
	WHtR	Healthy	21 (39.6)	34 (35.1)	1.216	0.579
		High	32 (60.4)	63 (64.9)		
	Lead	Normal	18 (34.0)	27 (27.8)	1.333	0.434
		Abnormal	35 (66.0)	70 (72/2)		
	Cadmium	Normal	50 (94.3)	85 (87.6	2.353	0.190
		Abnormal	3 (5.7)	12 (12.4)		

*Note:* Bold values indicate significant association between variables and occupation.

The final logistic regression model across occupational groups showed that Pb and Cd were not significantly associated with hypertension: Miners: (Pb; AOR = 1.034, 95% CI = 0.266 – 4.012, *p* = 0.962, Cd; AOR = 0.764, 95% CI = 0.268–3.912, *p* = 0.906). Artisans: (Pb; AOR = 2.830, 95% CI = 1.431–2.611, *p* = 0.631, Cd; AOR = 0.482, 95% CI = 0.088 – 2.638, *p* = 0.400). Laborers: Pb; AOR = 0.991, 95% CI = 0.426–2.309, *p* = 0.984, Cd; AAOR = 1.284, 95% CI = 0.476–3.463, *p* = 0.622). Farmers: (Pb; AOR = 0.602, 95% CI = 0.259 – 1.401, *p* = 0.239, Cd; AOR = 0.337, 95% CI = 0.083–1.364, *p* = 0.127) (Table 3).

**TABLE 3:** Logistic regression model for the association between anthropometric characteristics, heavy metals, and hypertension.

Occupation	Variable	Unadjusted OR	aOR (95% CI)	*p* value
Miners	Age ≥ 18 (adult)	1.077	1.076 (1.029–1.125)	**0.001**
	BMI (obese)	0.451	0.416 (0.093–1.860)	0.251
	WC (high)	1.552	0.699 (0.176–2.787)	0.612
	HC (high)	1.854	1.455 (0.470–4.504)	0.512
	WHR (high)	0.549	0.560 (0.156–1.628)	0.252
	WHtR (high)	1.032	1.185 (0.323–4.343)	0.798
	Lead (abnormal)	0.985	1.034 (0.266–4.012)	0.962
	Cadmium (abnormal)	0.731	0.764 (0.268–3.912)	0.906

Artisans	Age ≥ 18 (adult)	7.650	11.938 (3.349–38.621)	**< 0.001**
	BMI (obese)	2.185	1.692 (0.389–7.362)	0.483
	WC (high)	4.273	1.789 (0.208–1.540)	0.597
	HC (high)	17.043	2.500 (1.130–3.207)	**0.041**
	WHR (high)	1.885	1.111 (0.290–4.253)	0.878
	WHtR (high)	1.556	0.449 (0.102–1.978)	0.290
	Lead (abnormal)	1.273	2.830 (1.431–2.611)	0.631
	Cadmium (abnormal)	0.603	0.482 (0.088–2.638)	0.400

Laborers	Age ≥ 18 (adult)	6.395	5.300 (0.627–44.786)	0.126
	BMI (obese)	1.774	1.954 (0.181–21.113)	0.581
	WC (high)	2.380	3.427 (1.205–9.747)	**0.021**
	HC (high)	0.822	0.435 (0.174–1.087)	0.075
	WHR (high)	1.941	1.055 (0.446–2.945)	0.903
	WHtR (high)	0.900	0.917 (0.406–2.071)	0.836
	Lead (abnormal)	1.222	0.991 (0.426–2.309)	0.984
	Cadmium (abnormal)	1.122	1.284 (0.476–3.463)	0.622

Farmers	Age ≥ 18 (adult)	3.542	3.211 (0.838–12.313)	0.089
	BMI (obese)	1.108	1.030 (0.325–3.268)	0.960
	WC (high)	1.322	1.267 (0.421–3.810)	0.674
	HC (high)	1.386	1.315 (0.578–2.988)	0.514
	WHR (high)	1.455	1.681 (0.780–3.624)	0.185
	WHtR (high)	0.822	1.054 (0.426–2.637)	0.901
	Lead (abnormal)	0.750	0.602 (0.259–1.401)	0.239
	Cadmium (abnormal)	0.425	0.337 (0.083–1.364)	0.127

*Note:* Bold values indicate significant association with higher odds of hypertension.

## 4. Discussion

Exposure to heavy metals either occupationally related or, otherwise, has been reported to be associated with various negative health issues such as cancer, cardiovascular system–related disease such as hypertension, and neurotoxic effects. The present study showed possible association between heavy metals and anthropometric parameters across occupational groups and possible predisposition to hypertension. Furthermore, the logistic regression model showed that age predicted hypertension among miners and artisan. Additionally, circumferences predicted hypertension among artisans and laborers. The current study observed that age was strongly associated with hypertension among occupational groups exposed to heavy metals, suggesting that changes in body systems associated with natural aging process could contribute to hypertension amongst occupational groups.

In the present study, BMI and WC showed strong associations with hypertension among, miners, artisans, and laborers, suggesting that excess adipose fat around the waist region may be significantly associated with increased thickness levels of arterial intima, contributing to increased blood flow mechanics resulting in hypertension. Previous reports indicated positive correlation between BMI and WC and hypertension [29, 61, 62]. For instance, Mfuru et al. [63] observed association between WC and hypertension and further suggested that incorporating WC in cardiovascular risk assessment could enhance early detection and improve prevention efforts. Furthermore, the results showed correlation between WHtR and hypertension among farmers, which is consistent with previous reports of positive effect of WHtR on hypertension in cardiovascular disease studies that have been reported, alluding to the point that increased age and adipose tissue accumulations are precursors to hypertension [38, 64, 65].

Heavy metals are naturally existing substances widely distributed in the environment and commonly used in industry, households, agriculture, medicine, and other fields [28]. The increased concentration of heavy metals in the environment is in line with elevated levels detected in blood samples and may be a consequence of the expansion of mining activity in the area, which was currently observed in the present study. Furthermore, hypertension is an independent predictor of mortality and is becoming increasingly prevalent with rising urbanization and anthropogenic activities. As noted in the present, hypertension could be influenced by age, BMI, WHR, WHtR, Pb, and cadmium.

The accumulation of Pb and Cd in the human body promotes arterial atherosclerosis through mechanisms such as lipid peroxidation, vascular inflammation, endothelial dysfunction, and inhibition of nitric oxide [66]. The lead (Pb) levels observed in the current study exceeded the defined standard BLL < 10 μg/dL in adults [67], underscoring the need for regular biomonitoring of occupationally exposed workers. Furthermore, while the present study observed no significant association between Pb exposure and hypertension among occupationally exposed workers, nonetheless, the potential adverse effects in the local environment have been widely documented, as shown in Figure 2. For instance, Mailloux et al. [68] observed that Pb exposure promotes oxidative stress, impairs ATP production, and increases lipid production. Furthermore, ROS generated by metals could further disrupt mitochondrial function and impair the tricarboxylic acid cycle via oxidative phosphorylation, potentially contributing to hypertension [69]. This was in corroboration with the study of Faridah et al. [70] who observed strong association between Pb exposure and hypertension. The association observed between Pb exposure and hypertension among miners and artisans in this study support the concept that increased Pb concentration contributes to elevated hypertension risk, which is in agreement with Kisok and Park [25] who also observed positive association between higher blood concentrations of lead (Pb) and cadmium (Cd) and hypertension among adults in South Korea. Another study reported positive association between Pb and BP in children and adolescents [66].

**FIGURE 2:**
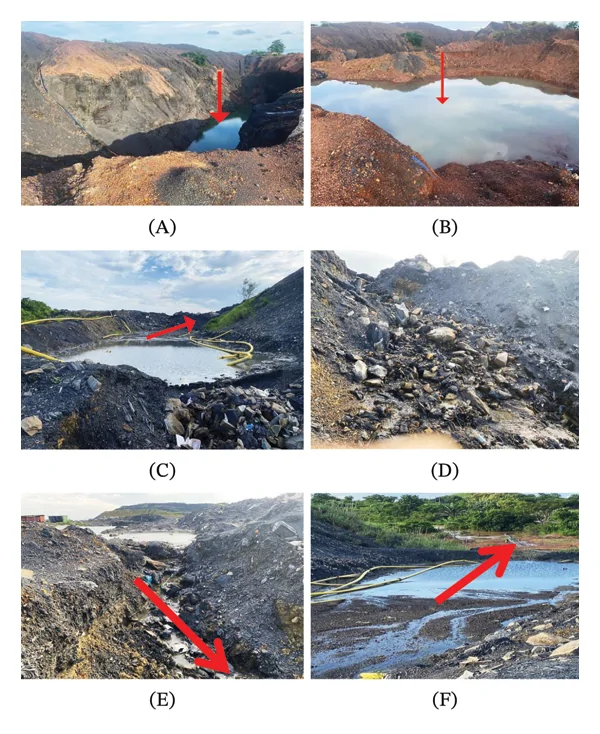
The mining site characterized by heavy metals. (A) and (B) represent the depth of the pit, while (C) indicates the heavy metals deposit and potential pathways for contaminated water to flow into the environment. (D) represents a large deposit of heavy metals containing lead and cadmium, while E and F represent pathways for the release of contaminated substances into the environment.

Cadium (Cd), a toxic element widely distributed in the environment, poses health risk to multiple organ systems and contributes to the development of hypertension [70]. As currently observed, Cd showed no significant association with hypertension among occupationally exposed workers. This could be attributed to level Cd within the level of measurable increase in the BP. Furthermore, the cross‐sectional design may have limited the ability to establish a temporal relationship between Pb and Cd exposure and hypertension. Unmeasured or residual confounding by factors such as lifestyle factors and duration of occupational exposure may also have influenced the observed association. In contrast, Hyun et al. [71] reported strong association between Cd and hypertension among smelting industry workers, while Satarug et al. [72] observed an association between Cd and hypertension among local residents of Thailand.

The current logistic regression analysis pinpoints age as predictor of hypertension in a model across occupational groups. The derived regression model, which included age as a predictor, showed that increasing age was significantly associated with higher odds of hypertension among miners (aOR: 1.076, 95% CI: 1.029 – 1.125) and artisans (aOR: 11.938, 95% CI: 3.349 – 38.621). Furthermore, anthropometric circumferences were significant predictors of hypertension among artisans (aOR: 3,427, 95% CI: 1.205 – 9.747) and laborers (aOR: 2.50, 95% CI: 1.130 – 3.207). Though many researchers have documented the roles of heavy metals and other anthropometrics like weight, height, BMI, and WC in understanding and predicting hypertension [28, 29, 66], in the current study, heavy metals and other anthropometries did not significantly contribute to the predictive models beyond the information already accounted for in the model specification; however, the importance of heavy metals contribution and relevance in occupational health and hypertension risk assessment remain crucial.

The strong association between age and hypertension in the final model regardless of sex may be attributed to age‐related arterial stiffening, reduced vascular elasticity, and changes in the body’s regulatory systems among occupationally exposed workers. These age‐related changes impair the heart’s ability to pump blood efficiently, leading to elevated SBP [72]. Primary advantage of this study is the ability to quantify both serum heavy metal concentrations and anthropometrics in a combined model, allowing the concurrent observation of multiple elements and their relationship with hypertension.

Several remediation strategies should be adopted, including phytodepuration, chemical immobilization, and bioremediation, to restore contaminated environment/workplace and enhance safety for both human health and ecological systems [73, 74]. Furthermore, easy access to tertiary care facilities with advanced diagnostic capabilities should be encouraged to avoid advanced toxicities of these metals. The data extrapolated from our study may be invaluable for medical administrators for developing guidelines on preventive measures aimed at improving working conditions and minimizing occupational exposure to heavy metals and associated health hazards. Although our study results did not show significant association between Pb, Cd, and hypertension, they were included as covariate in the logistic regression model to account for their potential influence on hypertension. After adjustment for these covariates, the associations between the other predictors and hypertension were evaluated. Periodic screening of workers occupationally exposed to lead (Pb) and cadmium is, therefore, recommended, particularly for the early detection of elevated BP and other potential health effects.

## 5. Conclusion

The study highlights the association between serum heavy metals, age, anthropometric determinants, and hypertension among occupationally exposed individuals. A significant association was observed between age and hypertension among occupationally exposed workers. Furthermore, age predicted the risk of hypertension across miners and artisans. Additionally, anthropometric circumferences predicted hypertension among artisans and laborers. Target interventions and routine screening programs could help minimize exposure‐related health risks among occupationally exposed workers. Individuals exposed to heavy metals poisoning (e.g., miners, artisans, laborers, and farmers) should regularly undergo a complete diagnostic assessment to monitor the blood concentration and enabling timely interventions and reducing the long‐term burden of hypertension‐related complications. In addition, the study provides some valuable insights for health policymakers, environmental epidemiologists, and researchers.

## 6. Limitations

The study focused on the effect of heavy metals and anthropometric characteristics on BP, without accounting for potential cofounding factors such as environmental, sociodemographic and economic, and physiology factors. The study focused on Pb, Cd, and anthropometries based on the prevailing environmental activities. Although the sample size was adequate for the study population, the generalizability of the findings may be limited. For instance, exposure duration, intensity, and historical exposure were not adequately characterized. Nevertheless, the current findings represent relevant determinants among hypertensive workers occupationally exposed to heavy metals. Further studies may investigate the effects of other heavy metals, environmental factors, and physiological and sociodemographic factors on BP in a broader population of occupationally exposed workers to enhance generalizability of the findings.

## Author Contributions

Study design: Godwin Chinedu Uzomba and Ejikeme Mbajiorgu; data collection: Collins Nwafor, Chibuike Ugah, Henry Ogbodo, Chukwuka Amaku, and Tamuno‐Ibelema Cookey‐Gam; data analysis: Godwin Chinedu Uzomba; data interpretation: Godwin Chinedu Uzomba and Godwin Sunday Obaje; preparation of the manuscript: Godwin Chinedu Uzomba and Ejikeme Mbajiorgu; literature search: Collins Nwafor, Chibuike Ugah, Henry Ogbodo, Tamuno‐Ibelema Cookey‐Gam, Godwin Sunday Obaje, Ken Onwe, and Godwin Chinedu Uzomba.

## Funding

The authors received no funding for the study.

## Disclosure

All authors approved to submit the journal.

## Ethics Statement

The local ethical committee of Alex Ekwueme Federal University Ndufu‐Alike (AE‐FUNAI/REC/VOL3/2023/201) approved the research protocol in accordance with the Declaration of Helsinki (2013).

## Consent

Informed consent was obtained from the participants. Subjects diagnosed with metabolic, cardiovascular, or respiratory diseases were excluded.

## Conflicts of Interest

The authors declare no conflicts of interest.

## Data Availability

Data used to support the findings of this study are available from the corresponding author upon reasonable request.
